# Neurofeedback in substance and non substance-related addictions: a mini review of current evidence and future directions

**DOI:** 10.3389/fpsyt.2025.1716390

**Published:** 2025-12-04

**Authors:** Bruna Sanader Vukadinovic

**Affiliations:** University College London Hospitals NHS Foundation Trust, London, United Kingdom

**Keywords:** neurofeedback, EEG, fMRI, addiction, substance use disorder, behavioral addiction, craving

## Abstract

Neurofeedback (NF) is emerging as a promising adjunctive intervention for substance use disorders (SUDs) and behavioral addictions. By providing real-time feedback on brain activity, NF enables individuals to self-regulate neural circuits implicated in craving, reward processing, and executive control. This mini review synthesizes recent evidence regarding the efficacy, mechanisms, and clinical applications of NF in addiction treatment. Addiction remains a major public health concern characterized by high relapse rates and limited long-term success from pharmacological and psychosocial therapies, underscoring the need for innovative approaches. Studies included in this review were identified through PubMed, Scopus, and Web of Science using the terms “neurofeedback,” “EEG,” “fMRI,” “addiction,” “substance use disorder,” “behavioral addiction,” and “craving,” with priority given to publications from 2019–2025. Two primary NF modalities have been examined: electroencephalography (EEG)-based and functional magnetic resonance imaging (fMRI)-based neurofeedback. EEG-NF, particularly the alpha–theta protocol, has demonstrated reductions in craving, anxiety, and relapse rates among individuals with alcohol and substance dependence. fMRI-NF enables targeted modulation of addiction-related brain regions, such as the insula and dorsolateral prefrontal cortex, with region-specific but variable clinical effects. Systematic reviews indicate that NF, especially EEG-based, reliably reduces craving and improves psychological outcomes, though methodological heterogeneity limits generalizability. Recent advances integrating NF with biofeedback and machine learning approaches suggest potential for personalized and multimodal interventions. Overall, NF represents a promising, neuroscience-informed adjunct in addiction treatment, warranting large-scale controlled trials to establish efficacy and optimize clinical application.

## Introduction

Neurofeedback (NF) is gaining recognition as a valuable complementary approach for addressing both substance use disorders (SUDs) and behavioral addictions. By offering real-time insights into brain activity, NF empowers individuals to regulate neural processes associated with addictive behaviors. This mini review presents a summary of the latest and most significant findings regarding the efficacy, mechanisms, and clinical application of NF for both substance-related and behavioral addictions. In **Table 1**, a summary of the relevant studies examining both substance-related and non-substance-related addiction applications is presented, highlighting the key findings that underpin this mini review.

**Table 1:** Summary of key studies investigating neurofeedback interventions in substance and behavioral addictions.

Study	NF type	Population	Target/Protocol	Sessions	Main outcomes	Limitations
Fathi et al. (2025) (1)	EEG/fMRI/fNIRS	SUD & Behavioral	Various	10–30	↓ craving, ↑ mental health	Heterogeneity of methods
Dave & Tripathi (2023) (4)	EEG	Alcohol use disorder	Alpha–theta	12	↓ craving, ↓ anxiety	Small samples, bias risk
Murphy et al. (2024) (2)	fMRI	SUD	Insula/DLPFC	4–8	Mixed changes in craving	Inconsistent findings
Fede et al. (2023) (5)	fMRI	Alcohol dependence	DLPFC & Striatum	4	↓ craving, region-specific effects	Short follow-up
Sanader Vukadinovic et al. (2024) (6)	fMRI	Alcohol dependence	Ventral attention network	3	↓ resting-state activity	Small sample
Tosti et al. (2024) (7)	EEG + Biofeedback	Nicotine addiction	Integrated	10	↓ withdrawal, ↑ mood	Narrative review
Unterrainer (2025) (8)	EEG	Behavioral addictions	Self-regulation	Variable	↑ emotional insight	Few controlled studies

Addiction, encompassing both substance-related and behavioral forms, remains a major public health challenge with high rates of relapse and significant morbidity. Traditional treatments, including pharmacotherapy and psychosocial interventions, often yield suboptimal long-term outcomes, prompting the search for novel adjunctive therapies. Neurofeedback, a form of biofeedback that provides individuals with real-time information about their brain activity, aims to enhance self-regulation of neural circuits implicated in craving, reward processing, and executive control (1–3, 8).

The studies discussed in this review were identified through searches in PubMed, Scopus, and Web of Science using combinations of the terms “neurofeedback,” “EEG,” “fMRI,” “addiction,” “substance use disorder,” “behavioral addiction,” and “craving.” Recent publications (2019–2025) were prioritized. Both clinical and experimental studies investigating neurofeedback interventions for substance or behavioral addictions were considered. Exclusion criteria comprised animal studies, non-peer-reviewed reports, and studies focusing exclusively on other psychiatric disorders without addiction-related outcomes.

The two principal modalities of neurofeedback in addiction research are electroencephalography (EEG)-based and functional magnetic resonance imaging (fMRI)-based neurofeedback. EEG-NF has been studied since the late 20th century, with early reports suggesting reductions in craving and improvements in psychological well-being among individuals with alcohol and other substance use disorders (1, 4). More recently, fMRI-NF has enabled targeted modulation of specific brain regions, such as the insula, anterior cingulate cortex, and dorsolateral prefrontal cortex, which are central to the neurobiology of addiction (2, 3, 5, 6, 9, 10).

Systematic reviews and meta-analyses have begun to clarify the potential and limitations of neurofeedback in addiction. Fathi et al. identified 32 studies (18 EEG-NF, 11 fMRI-NF, 3 fNIRS-NF) and reported that neurofeedback, especially EEG-NF, consistently reduced drug craving and improved some mental health outcomes, though heterogeneity in protocols and outcome measures remains a challenge (1). Murphy et al. reviewed 16 fMRI-NF studies in SUDs, highlighting early but inconsistent evidence for changes in brain function and craving, and emphasizing the need for more rigorous, placebo-controlled trials (2). Dave and Tripathi systematically reviewed 20 studies on neurofeedback for alcohol use disorder (AUD), finding temporary reductions in craving, anxiety, and relapse rates, but noting that most studies were small and at moderate risk of bias (4).

Recent advances include the use of individualized whole-brain classifiers in fMRI-NF for smoking craving, which have demonstrated improved accuracy and potential for personalized neuromodulation strategies (10). Integrated neurofeedback and biofeedback approaches have also shown promise in nicotine addiction and other conditions, suggesting synergistic effects on withdrawal symptoms and psychiatric comorbidities (7). In behavioral addictions, such as gambling and internet gaming, the literature is less robust but indicates potential benefit, particularly with EEG-NF targeting self-regulation and reward processing circuits (1, 8).

## Discussion

The clinical application of neurofeedback in addiction is supported by a growing, though still preliminary, body of evidence. EEG-NF, especially the alpha-theta protocol, has been the most extensively studied modality in SUDs. Multiple studies have reported reductions in craving, anxiety, and depressive symptoms, as well as improvements in abstinence rates, following EEG-NF interventions in alcohol, opioid, and stimulant use disorders (1, 4). The alpha-theta protocol, which aims to increase theta and alpha power while reducing high-frequency beta activity, is hypothesized to facilitate relaxation and enhance access to subconscious processes, potentially supporting recovery from addiction (1, 4).

Comparative analysis of existing NF protocols suggests that certain parameters may underlie stronger clinical outcomes. EEG-based alpha–theta protocols, for instance, appear particularly effective in alcohol and opioid dependence, likely due to their emphasis on enhancing relaxation and emotional processing through increased alpha and theta activity. By contrast, fMRI-NF protocols demonstrate greater precision in targeting craving-related regions such as the insula and dorsolateral prefrontal cortex, offering region-specific modulation but at substantially higher cost and lower accessibility. Evidence indicates that outcomes are influenced by neuroanatomical targets (e.g., insula vs. prefrontal control networks), session number (≥10 sessions generally producing more stable effects), and participant characteristics—particularly motivation, baseline craving intensity, and comorbid anxiety or depression. These factors together may explain the variability in treatment success across studies.

The heterogeneity of EEG-NF protocols, session numbers, and outcome measures complicates direct comparisons and meta-analytic synthesis. Some studies have reported sustained benefits at follow-up, while others have found only transient effects. The lack of standardized protocols and the frequent absence of active control conditions or blinding further limit the strength of the evidence (1, 4).

fMRI-NF offers the advantage of targeting specific brain regions implicated in craving and self-control. Studies have demonstrated that individuals with SUDs can learn to downregulate activity in the insula, anterior cingulate cortex, and dorsolateral prefrontal cortex, with associated reductions in self-reported craving (2, 3, 5, 6, 9, 10). For example, Fede et al. found that greater downregulation of the dorsolateral prefrontal cortex and striatum during fMRI-NF was associated with larger reductions in alcohol craving, and that multi-region-of-interest feedback outperformed support vector machine-based approaches (5). Sanader Vukadinovic et al. reported that three sessions of fMRI-NF in alcohol-dependent patients led to significant reductions in resting-state activity within the ventral attention network, suggesting a shift toward greater independence from external stimuli and potentially improved cognitive control over craving (6).

Despite these promising findings, the clinical impact of fMRI-NF remains uncertain. Karch et al. conducted a double-blind, placebo-controlled trial of fMRI-NF as an adjunct to standard treatment in alcohol dependence and found that while neurofeedback modulated addiction-related brain responses, it did not result in clinically superior abstinence rates at three months compared to sham feedback (9). Murphy et al. similarly concluded that evidence for changes in brain function and craving following fMRI-NF is early and inconsistent, and that more rigorous, repeated-measures designs with placebo controls are needed (2).

Recent methodological innovations include the use of individualized whole-brain classifiers for fMRI-NF, which have demonstrated higher accuracy in distinguishing craving from non-craving states and greater potential for personalized neuromodulation strategies (10). Kim et al. showed that individual-level classifiers outperformed group-level classifiers in modulating smoking craving-related brain responses, and that accuracy improved across repeated neurofeedback runs, supporting the feasibility of learning individualized strategies for craving regulation (10).

Integrated neurofeedback and biofeedback approaches have also been explored, particularly in nicotine addiction. Tosti et al. reviewed studies combining neurofeedback with other biofeedback modalities and found evidence for reductions in psychiatric symptoms, anxiety, depression, and withdrawal symptoms, as well as improvements in self-esteem among smokers (7). These findings suggest that multimodal interventions may offer synergistic benefits, though further research is needed to determine their superiority over single-modality approaches.

In non-substance (behavioral) addictions, such as gambling and internet gaming, the evidence base is less developed but indicates potential benefit. Fathi et al. identified several studies reporting improvements in self-regulation and reward processing following EEG-NF in behavioral addictions, though the number of studies remains small and methodological rigor is variable (1). Unterrainer highlights the potential for EEG-NF to foster neurophysiological regulation and emotional insight in individuals with behavioral addictions, particularly when combined with psychodynamic therapy (8).

Mechanistically, neurofeedback is thought to facilitate neuroplasticity and enhance self-regulation of brain circuits involved in craving, reward, and executive control. fMRI-NF studies have demonstrated that successful downregulation of craving-related brain regions is associated with greater reductions in subjective craving and improved abstinence outcomes (5, 6, 9, 10). Resting-state fMRI data suggest that neurofeedback may restore functional connectivity within networks implicated in addiction, such as the ventral attention and default mode networks (6, 9). EEG-NF may promote relaxation and access to subconscious processes, supporting recovery through mechanisms distinct from those targeted by fMRI-NF (1, 4).

Despite these advances, several challenges remain. The heterogeneity of neurofeedback protocols, small sample sizes, and lack of standardized outcome measures limit the generalizability of findings. Many studies lack active control conditions or blinding, increasing the risk of bias. The optimal number and duration of neurofeedback sessions, as well as the most effective feedback modalities and target brain regions, remain to be determined (1–6, 9, 10).

Furthermore, the clinical significance of neurofeedback-induced changes in brain activity is not always clear. While some studies have demonstrated associations between neural modulation and reductions in craving or relapse rates, others have found no significant differences in clinical outcomes compared to standard care or sham feedback (2, 4, 9). Long-term follow-up data are sparse, and the durability of neurofeedback effects remains uncertain.

## Conclusion

Future research should prioritize large-scale, randomized controlled trials with standardized protocols, active control conditions, and long-term follow-up to clarify the clinical utility of neurofeedback in addiction. The integration of neurofeedback with established psychosocial and pharmacological interventions may enhance treatment outcomes, particularly for individuals with refractory or comorbid conditions. Advances in neuroimaging and machine learning may enable more precise targeting of neural circuits and personalized feedback strategies, further improving the efficacy of neurofeedback interventions (10).

Future research should also explore integration of neurofeedback with emerging digital and virtual reality (VR)-based interventions, which could enhance engagement and generalization of self-regulation skills to real-world contexts. The application of artificial intelligence and adaptive algorithms may enable real-time personalization of feedback parameters, improving learning efficiency and treatment outcomes. Furthermore, systematic evaluation of cost-effectiveness, scalability, and clinical feasibility in community and outpatient addiction services is needed to inform implementation. These directions will determine whether neurofeedback can transition from a promising research tool to a routine clinical option for addiction treatment.

In summary, neurofeedback represents a promising adjunctive intervention for substance and non-substance-related addictions, with preliminary evidence suggesting improvements in self-regulation, craving reduction, and relapse prevention, though further large-scale, rigorously controlled trials are required to establish its efficacy and clinical applicability.
